# Supplementary material for: PEST sequences from a cactus dehydrin regulate its proteolytic degradation
Source: PeerJ. 2019 May 14;7:e6810. doi: 10.7717/peerj.6810 (PMC6524633; doi:10.7717/peerj.6810)
Supplement: Table S1 [file peerj-07-6810-s003.pdf]

Potential PEST sequence occurrence in DHN subclasses

SK-Type

Specie	Access number/Name	PEST score	Position	Protein size	Sequence
Amborella trichopoda	AtrDHN-SK	6.65<br>0.87<br>12.19	162-177<br>5-17<br>201-212	212	MADEHTTSVVPESGDGRVEVHERGLFGHHHPETPEQKQEEALASKFEEVYVSEEKKEEKEKDEEKKGSLLEKLHRSGSSSSSSSDEEEAEEWKKKKKERKGLKEKIKEKLSGGSEAVKEKAGEGAPHCAGAEHAHEKEKKGLPEKIKEKLHGGGGYGAPQHPVEAPESAPQTSAPHPEGQSHEEKKGFLEKIKEKFSGYHPNSGEETTPSH
Amborella trichopoda	AtrDHN-SK2	ND		148	MAEYRGDERYQKHEHGYEQGQPGYYGGTVTGTGTGAGTGAGTGTGGVMLGTTGAGMMHGTQPEQQKLHRSNSSSSSSSEDDGMGGRRKKKGLKEKIKEKLPGGHKTTEGQGHEAGAGVHGHEHEHEKKGIIEKIKEKLPGHHKDQLNA
Aquilegia coerulea	AcDHN2-SK2	4.15	125-136	233	MAEQQPQTTVEGAEIKDRGMFDFLSKKKEEEKPQEAEVLATELENVHVSEPVHDHHVAEPVEVKKEEVDEEKKPSLLEKLHRSDSSSSSSSSEEEVEEDGQKIKRKKNKGLKEKIKEKISPEEKKVEEDTTVPIEKFDDEVGVVSHEGEVKHPEEKKTFMEKIKEKLPGGNKKTADHESASTPDAIVLPPPAAPVHEEPKEKKGILEKIKEKLPGYHKTEDEKKETTTTTEH
Arabidopsis halleri	Araha.<br>12526s0002.1_SK2	ND		106	MQSSDEEAGENGEKKEKEKKNKKKKEVVADQCETEEKNKEKLPAGIGHEDGKEKGFMEKMKDKLPGGHHGKPEAEPHNDKGKEKGFMEKIKEKLPGHTNDEKKKET
Arabidopsis halleri	Araha.23419s0001.1<br>(ERD10)	0.95<br>11.33<br>4.96<br>2.52	202-224<br>6-21<br>36-49<br>160-173	258	MAEEYKNNVPEESSTTTPEVKERGMFDFLKKKEEVKPQETTTLASEFEHKTQISEPEPFVAKHEEEEQIKEQKPTLLEKLHQKHEEEEENKPSLLDKLHGSNSSSSSSSDEEGEDGEKRKKKKEKKKIVEGDVKTEEEENQGVMDKIKEKFPYGEKPEGHDDVPEITTMPAPHSVEDKKPEEEEKKGLMDKIKEKLPGHSKKPEDSQVVNSAPLVETATPIAEHPEEKKGFMDKIKEKLPGYHAKTSGEEEKKDKESL
Arabidopsis halleri	Araha.3199s0027.1<br>(ERD14)	12.07<br>4.52	43-56<br>58-69	190	MAEEIKNVHEQEVPKVATEESSSAEVTDRGLFDFLGKKKGETKPEETTIDSEFEQKVHISEPEPAPEVKHESLLEKLHRSDSSSSSSSEEEGEDGEKRKKKKEKKKPVAEVEVKTEEEKKGFMEKLKEKLPGNSKKPEDGSAVAAAPVVVPPPVEEAHPAEKKGILEKIKEKLPGYHPKTTVEEEKKDKE
Arabidopsis halleri	Araha.9031s0001.1<br>(COR47)	4.53<br>4.40<br>4.40<br>11.04	153-175<br>199-217<br>16-32<br>46-59	260	MAEEYKNNVPEHETPKVATEESPATTTEVTDRGLFDFLGKKEEEVKPQETTTLEPEFDHKAQNSEPELAAEHEEVKENKITLLEELQEKTEEDEENKPSVIEKLHRSNGSASSSSDEEGEEKKEKKKKIVEGEEEKKGLAEKIKEKLPGHHDKKPEDDVPVSTTIQVPVSESVVEHDQPEEEKKGPVQKIKEKLPGHHDKTAEDSPAVTSTPLVVTEHPVEPTTEHPEEKKGILGKIKEKLPGYHAKTTEEEEKKVKETDA
Arabidopsis lyrata	AL1G33370.t1 (COR47)	6.70<br>7.91<br>12.64<br>10.60	153-175<br>199-217<br>16-32<br>46-59	264	MAEEYKNNVPEHETPKVATEESPATTTEVTDRGLFDFLGKKEEEVKPQETTTLESEFDHKAQISEPELAAEHEEVKENKITLLEELQEKTEEDEENKPSVIEKLHRSNSSASSSSDEEGEEKKEKKKKIVEGEEEKQGLAEKIKEKLPGHHDKKPEDDVPVSTTIPVPVSESVVEHDQTEEEKKGLGEKIKEKLPGHHDKTPEDSPAVTSTPLVVTEHPVEPTTEHPVEHPEEKKGILEKIKEKLPGYHAKTSEEEEKKVKETDD
Arabidopsis lyrata	AL1G33380.t1 (ERD10)	1.45<br>11.33<br>4.96<br>0.50	200-222<br>6-21<br>36-49<br>50-62	256	MAEEYKNNVPEESSTTTPEVKERGLFDFLKKKEEVKPQETTTLASEFEHKTQISEPEPSVAKHEDEELKEHKPTLLEKLHQKHEEEEENKHGLLDKLHRSNSSSSSSSDEEGEDGEKRKKKKEKKKIVEGDVKTEEEENQGVMDKIKEKFPYGEKPEGHDVPVVTTMPAPHLVDDKKPEEEEKKGLMDKIKEKLPGHSKKPEESQVVNTAPLVETATPIAEHPEEKKGFMDKIKEKLPGYHAKTSGEEEKKDKESL
Arabidopsis lyrata	AL2G36240.t1_(ERD14)	12.07<br>4.52	43-56	106	MAEEIKNVPEQEVPKVATEESSSAEVTDRGLFDFLGKKKDETKPEETTIDSEFEQKVHISEPEPAPEVKHESLLEKLHRSDSSSSSSSEEEGEDGEKRKKKKEKKKSVAEVEVKTEEEKKGFMEKLKEKAAAPVEEAHPAEKKGILEKIKEKLPGYHPKTTVKEEKKDKE
Arabidopsis lyrata	AL7G10280.t1_SK2	ND		164	MADHPRSSEQQEADVAASKGCGMFDFLKKKPEDVHSSENAGVTKEPKEEEKPSLAERLHLSDSSSSDEEAGENGEKKEKENKKKNKKEVVAAQCETEEKLPAGRGHEDGKEKGFMEKMKDKLPGGHHGKPEAEAHNDKGKEKGFMEKIKEKLPGHTNDETKKET
Arabidopsis thaliana	AT1G20440-<br>SK3(COR47)	5.35<br>18.89<br>2.40<br>10.60<br>6.02	153-176<br>12-32<br>201-218<br>46-59<br>218-230	265	MAEEYKNNVPEHETPTVATEESPATTTEVTDRGLFDFLGKKEEEVKPQETTTLESEFDHKAQISEPELAAEHEEVKENKITLLEELQEKTEEDEENKPSVIEKLHRSNSSSSSSSDEEGEEKKEKKKKIVEGEEDKKGLVEKIKEKLPGHHDKTAEDDVPVSTTIPVPVSESVVEHDHPEEEKKGLVEKIKEKLPGHHDEKAEDSPAVTSTPLVVTEHPVEPTTELPVEHPEEKKGILEKIKEKLPGYHAKTTEEEVKKEKESDD
Arabidopsis thaliana	AT1G20450-<br>SK3(ERD10)	7.32<br>4.96<br>1.34	204-230<br>43-56<br>16-28	260	MAEEYKNTVPEQETPKVATEESSAPEIKERGMFDFLKKKEEVKPQETTTLASEFEHKTQISEPESFVAKHEEEEHKPTLLEQLHQKHEEEEENKPSLLDKLHRSNSSSSSSSDEEGEDGEKKKKEKKKKIVEGDHVKTVEEENQGVMDRIKEKFPLGEKPGGDDVPVVTTMPAPHSVEDHKPEEEEKKGFMDKIKEKLPGHSKKPEDSQVVNTTPLVETATPIADIPEEKKGFMDKIKEKLPGYHAKTTGEEEKKEKVSD
Arabidopsis thaliana	AT1G76180-<br>SK2(ERD14)	6.83	42-55	185	MAEEIKNVPEQEVPKVATEESSAEVTDRGLFDFLGKKKDETKPEETPIASEFEQKVHISEPEPEVKHESLLEKLHRSDSSSSSSSEEEGSDGEKRKKKKEKKKPTTEVEVKEEEKKGFMEKLKEKLPGHKKPEDGSAVAAAPVVVPPPVEEAHPVEKKGILEKIKEKLPGYHPKTTVEEEKKDKE
Arabidopsis thaliana	AT4G38410-SK2	ND		163	MADHPRSTEQQEADAAASKGCGMFDFLKKKPEDVHSSENARVTKEPKEEEKPSLAERFHLSDSSSSDEEAGENGEKKEKKKKKKNEVAEDQCETEEKIPAGIGHEDGKEKGFMEKIKDKLPGGHNGKPEAEPHNDKAKEKGFMEKIKEKLPGHTNDEKKKET
Brachypodium distachyon	BdDHN1-SK3	12.51<br>17.87	204-219<br>158-169	252	MEDERNTQSHQAADQVEVTDRGLFDKFIGKKKEEEDKKQEEVLVTGMEKVSVEEPEVKEEEVHQDGEKKESLFTKLQRSSSSSSSSSDEEEEVIDDNGEVIKRKKKKGLKEKIKEKLPGHKDTEGEHKTAAPTPGPPVPSHHDDDGAVVVEKIDGEVKTEAPPAPEEEKKGFLEKIKEKLPGGHKKPEDTAAVPVTHGAPAPVHTPGPAATEEVSSPEKKGILGKIMDKLPGYHKTPGEEDKAAAGEHKTTA
Brachypodium distachyon	BdDHN2-SK3	6.71<br>10.00<br>3.94	122-140<br>158-169<br>205-221	254	MEDERSTQSHQGGEADQVEVTDRGLFDKFIGKKKEEEDKKQEEVLVTGMEKVSVEEPEVKEEEHQDGEKKESLFTKLQRSSSSSSSSSDEEEEVIDDNGEVIKRKKKKGLKEKIKEKLPGHKDTETAAPTPAPPAPVVTHGGHHDDGAVAVEKIDEVKTEAPVAPEEEKKGFLEKIKEKLPGGHKKPEEAAAVPAVTHAAPAPVHAPAVPAATEEVSSPEKKGILGKIMDKLPGYHKTPGEEDKAAAGEHKTTA
Cajunus cajan	CajDHN-SK	ND		126	MAEAQIRDQLGNPIPLTDQFGNPVKLTDENGNPVVLTGVATTLTGTVPDLLPTQARNNETDLARSSSTTSTSSSSSSSSSSEDDEEQHAEITTASHPTVTTISHPEPEKKGLLEKIKEKLPGHLNQ
Cajunus cajan	CajDHN-SK2	4.44<br>11.45	8-22<br>156-171	216	MAEEPQNKYETSQPSEVEVNDRGVFDFLGKKKEEDKPQEEVIVTEFENKVTVSGEADKKQEEGEKKPSLLEKLHRSDSSSSSSSEEEGEDGEKKKKKKKEKKGLKEKIEEKIGGDHHKEEDTSVPVEKVEVVDAPHPEEKKGFLEKIKEKLPGQKKEEATPPPPVAASSDHGEGAHHHEGAEAKEKKGILEKIKEKLPGYHPKTEEEKEKESGAH
Capsicum annum	CaDHN24-SK3	11.84<br>12.02	162-181<br>9-26	219	MADQYEHNKPSVEETTTGANVESTDRGLFDFLGKKEEEKPTHAHEEQAISSEFGEKVKVGEEEHKEEEKKKEEKKLHRSSSSSSSSSDEEEEVVGEDGQKIKKKKKKGLKDKMKDKISGDHKEEDKKTEDTSVPVEKYEETEEKKGFLDKIKDKLPGGGQKKTEEAAAPPSPPPPPAVAEHEAEGKEKKGFLDKIKEKLPGYHSKTEEEKEKEKEAASH
Citrus x paradisi	CpDHN-SK3	2.98<br>4.68	12-27<br>189-201	234	MAEEIKKQQKSHEYEPSVGTEGAVETKDRGMLDFLGKKEEEKPQHHDQEVIATEFEKVHVSEPQPKVEEHRKEEKEEEKKPGFLDKLHRSTSSSSSSSDEEEGDDEEKKKKKKEKKGLKEKLKEKISGEKEEDTTVPVEKLDDVHAPHHQEEAHPEEKKGFLNKIKEKLPGQQKKPGDHQVPSPPAAEHPTSVEAPEAEAKEKKGILEKLKEKLPGYHPKSEDEKDKDKETAAH
Coffea canephora	CcaDHN-SK3	4.44<br>9.17	121-132<br>166-190	227	MAEYDQSNIKVEEGSAVEATDRGLFNLGKKEEVKKCDQGQAISAEFDEKVRVSEPDKEEGKKHGGLLEKLHRSGSSSSSSSEEEVEEGGEKKKKKEKKGLKDKIKEKISGDKKDEEKVEKCEEDTSIPVEKYAEPAHADAAHEPEEKKGFLDKIKEKLPGGGQKKTEEVAAAAPPPPAECTATEGEAKDKKGFLDKIKEKLPGYHPKTEEEKEKEKEEKEAGCH
Hordeum vulgare	DHN10-SK	ND		295	MEYQGQHGNAANRVDQHGNPVAALGATAVTGPPTGGQLQPGREEHKTRGILHRRSSSSSSSSSEDDGMGGRRKKGIKEKIKEKLPGARKQTYGQPAAPAGMTGAGATGGPHYVQPAPAGTGVHGTTATTGTYVQPAPAGTGAHGTTATTGTYGQPAPAGMTGTGAHGTTATGEKKGMKDKIMEKLPGGHKNGQHTMPSAGAYGQPGMTAAGMDGNTTTGGGYGGQPGHAGTGTHGHATTGGAYDHQGNPGMTGTGAHGTTATGGAYNQQGHAGLAGTGEK KGIMDKIKEKLPGQH
Hordeum vulgare	DHN8-SK	2.63<br>12.28	204-219<br>157- 169	255	MEDERSTQSYQGAEADQVEVTDRGLLGNLLGKKKEEEDKKKEEELVTGMEKVSVEEPEVKEDGEKKETLFSKLHRSSSSSSSSSDEEEEEVIDENGEVIKRKKKKGLKEKLKEKLPGHKDNEAEHVTGLPAPMAPASVQTHHDTDVVVEKIDGDAKTEATPAVPEEEKKGFLEKIKEKLPGGHKKPEDAAAVPVTHAAPAPVHAPAPAAEEVSSPDAKEKKGLLGKIMDKLPGYHKTGEEDKAAAPSGEHKPRA
Lycopersicum	LyDHN-SK2	7.09	155-172	206	MADQYEQNKASVEETVGTNVEATDRGLFDFIGKKEEEKPSHAHEEEAISSELSEKVNVSEEVEPKEEEKKLHRSNSSSSSSSDEEEEIGEDGQKIKKKKKKGLKDKIKEKISGDHKEESKAEDTSVPVEKYEETEEKKGFLDKIKEKLPGGGHKKTEEVAAPPPPPPAAVEHEAEGKEKKGFLDKIKEKLPGYHSKAEEEKEKED
Malus domestica	MdDHN8-SK3	2.25<br>3.22<br>20.14	54-71<br>230-247<br>184-195	284	MAEEYNKSQEHEYERKTGDYEEGSGAGETKDRGLFDFLGKKAEEKPTSYQHEEKPASYQEEEVIVTEFDEKAKISDHHEALAPDQYTSSYSKVEEDEKKHENLLQKLHRSNSSSSSSSDEEEDEEKKQRKEKKGLKDKIKEKISGDDHKEEGYHKEDTAVPVEKVYEEEHHHQAPAPVVHHHEEPTDYPTEEKKGFLEKIKEKLPGHEKTEEVPVGAASYEQQSHDHHATEPPVVASYEAGEEPKEKKGIMEKIKEKLPGYHSKTEEDHKDIKEKEKDTPSY
Malus domestica	MdDHN9-SK3	0.91<br>28.65	223-240<br>177-188	277	MAEEYNKKSDEHEYERKTGDYEEGSGAGETKDRGLFDFLGKKEEEKPTPYQQGDQVNVAEFDEKVKISDHHDQHASSYNKVEEEKDEKKHETLLQKLHRSESSSSSSSDEEEDEEKKKRKKEKKGLTDKIKEKISGDEHKEEGYHKEEDTAVPVEKVYEEEHHHPAPAPAPVVHYHEEPTDSPTEEKKGFLEKIKEKLPGHKKTEEVPVGAASHEQHSDDKHAAEPPVAASYEAGEEPKEKKGILEKIKEKLPGYHSKPEEDHKDIKEKEKDTPSY
Musa acuminata	MaDHN-SK	ND		130	MAEEHHKAVESGEEVEVQDRGLFDFLGKKKEEEKTEECHEEVLVSGVEKIHLEEAGKEEDKKEGLLEKLHRSHSSSSSSSSDDEEEDGGENKEKKKKKKGLKEKIKEKLCGEKKEGEEEAKRRRVSWGS
Musa acuminata	MaDHN-SK	ND		96	MPATQGYGVGAGAAGKEQHHGGGGITGMLHRSGSSSSSSSEDDGQGGRRKKKGLKQKIKEKLPGGHKEHESSVEKQHEKKGFVEKVKEKMPGHHKE
Musa acuminata	MaDHN-SK2	2.66	164-179	220	MAEEHNKAMESGEEVAVQDRELFDFMEKKKDEERKECHEEEEVLVTGVEKVQIEEGEKANEEEKMGLLEKLHRSHSSSSSSSSDDEEEAEGENKEKKKKKKGLKEKIKEKLVCEEEAKKPEVDEGTAVVVEKMQEDTVKVEATPPEQEKGLLEKIKEKLPGHKKPADEAPAPPSPCAGHGEEHERDEGKEKKGILGRIMEKLPGYHKTEEKEGEKKSPSK
Opuntia streptacantha	OpsDHN1-SK3	3.80<br>8.06	146-166<br>237-248	248	MAEEHQKGDNVNVESSDRGLFDFMKKNKESGDEKKDQEEVIATEFHHKVEVSKEDKHDENKEGGLLHKLHRSDASSSSSDEEEGDDEDKKRRKKEKKKEKKGLKEKIKEKLPGHHKEQEEEQEDKQKDHHHHHHDEEDTNIAIEKIHVEEVIYSEPSYPAPAPPPPHLEAEGEKKGLLEKIKDKLPGQHKKAEAEHEVVPTATATVAEGEAQEKKGFLDKIKEKIPGFHFKAPEEDKKDVECDQPPSST
Oryza sativa	OsDHN1-SK3	7.14<br>14.84	213-238<br>238-252	290	MEDERNTESHQGGEAAEQVEVKDRGLFDNLLGRKKDDQPEEKKHEEELVTGMEKVSVEEPKKEEHHAEGEKKESLLSKLHRSSSSSSSSSDEEEVIDDNGEVVKRKKKKGLKEKIKEKLPGHKDHAGEHAPPPAATGFPAPAPPASVVTAAPTPAPAPVVTHGDHHHDTAVPVEKIEGDHAKTEATLPRAPEEEKKGFLDKIKEKLPGGHKKPEDATAVPPPAASPAAPATTPAPAHPPPATEEVSSPDGKEKKGILGKIMEKLPGYHKGSGEEDKTAA
Populus canadensis	Peudhn1-SK2	2.52	39-52	225	MAEGNKSHEYETKVGEESGAVETKDRGLFDFLGKKEEEKPQEEVIATDFEEKLQVSEPETKVEEEHKKKEEEEKKPTLFEKLHRSGSSSSSSSDEEEGDDEEKKKKKKEKRSLKEKMKISGEKGEEKEHEDTSVPVEVVHTETPHEPEDKKGFLDKIKEKLPGHKKADEVPPPAPEHVSPEAAVSHEGDAKEKKGLLEKIKEKIPGYHPKTEEEKEKEKESASQ
Populus trichocarpa	PtrDHN-3-SK2	3.84	39-52	226	MAEENKSHEYETKVGEESGAVETKDRGLFDFLGKKEEEKPQEEVIGTEFEEKLQVSEPETKVEEEHKKKEEEEKKPTLFEKLHRSGSSSSSSSDEEEGDDEEKKKKKKEKRSLKEKMKISGEKGEEKEHEDTSVPVEVVHTETPHEPEDKKGFLDKIKEKLPGHKKADEVPPPAPEHVSPEAAVSHEGDAKEKKGLLEKIKEKLPGYHP KTEEEKEKEKESASQR
Prunus pérsica	PpeDHN1-SK2	ND		182	MADHYPKETKESQSPTKVGEEGQGCGMFDFLKKKENEKPHQEQEQHKHTLAEKLHHHGGDSSSSSSDEEGGEKKKKGLKGKIKEKKSGKKEEGDAYHASVPVEKTHDIENGAPHADDKKGFIEKIKEKLPGQQKEAEHGAHAEYHAMDDHSHEAEPKKGILEKIKDKLPGGHKNEEEKPKEY
Prunus pérsica	PpeDHN2-SK2	0.37	184-198	254	MAEEYNKSQDRGYEGKAGEYEEGSGARAAECGEIKDRGLFDFLGKKEAEKPQEEVIVTEFEKVKVSDHEAPHPHHHEPESYKVEQEEDKEKKHGSLLEKLHRSDSSSSSSSDEEEGEGGEKKKKKKEKKGLKEKIKEKICGDHDQKVEDTAVPVEKIYEEPTHEEKKEEEKKGFLEKIKEKLPGQQKKPEEIPASYDDQQCHAQHAEPAEPAGVGCEPKEKKGILEKIKEKIPGYHPKTEEEKEAIKEKEKETSSY
R.catawbiense	RcDhn5-SK	1.80<br>24.98	170-199<br>224-235	240	MAEQPDHHQHHHHVEEKSGECGGAGKTGEVPIETADRGLFDFTAVKQKEECCEEIKTTHHVEEQDEVIGAEFDKLHVSEPEHKEEEKKGSLLEKFHRSDSASSSSSSDEEEGEEKKEKKKKKGLKEKKEKHEEDTNVPIEKYEEEAVAQPEEKKGFLDKIKEKLPGQHKKTEEAAVAPPPPPPVVVECYAAEESSQVGHEADQPKEKKGFLEKIKEKIPGYHPKSPTSSPSEEEKEKED
Setaria itálica	SitDHN2-SK2	17.43	194-205	290	MEDERNTQLHHQGGEAQEKAADQVEVKDRGILDTLLGRKKPEDQEKKQEEELVTGMEKVTVAEPEKHEHKKEEHEAGEKKESLLAKLHRTSSSSSSSSDEEEVIDENGEIVKRKKKKGLKEKIKEKLPGHKDHAEGEHHTAVPAPAPAPVETHAYKEDDHKPYVPAPAPPPVETHVHHHDHAVVVQKIEDDAKTDAPPAPEEEKKGLLDKIKEKLPGGHKKPEDAAGAPAVHAPAPTPHTEDVSSPDGKEKKGLLGKIMDKIPGYNKGSGEEDHKAAGAAAGEHKTTSS
Setaria itálica	SitDHN3-SK2	ND		138	MEHQGQHGHATNQANEYGNPVAAGHGATGVGAAGDQVQPMRDDHKTDGLLRRSGSSSSSSSEDDGMGGRRKKGIKEKIKEKLPGGNKDSTGQQHTTTGGAVGQQGHAGATGTGAHGTEGTGEKKGLMDKIKEKLPGQH
Setaria itálica	SitDHN5-SK2	ND		138	MEYQGQHGHATNQANEYGNPVAAGHGATGVGAAGDQVQPMRDDHKTDGLLRRSGSSSSSSSEDDGMGGRRKKGIKEKIKEKLPGGNKDSTGQQHTTTGGAVGQQGHAGATGTGAHGTEGTGEKKGLMNKIKEKLPGQH
Stipa purpurea	SpDHN1-SK3	6.19<br>17.87	217-232<br>171-182	271	MEDERNTQSYQGEADQVEVKERGLFDSLLGEKKQEEDKKQEEVLVTGMEKVSVEEPEVKEEEHEGGEKKESLLSKLQRSDSSSSSSSDEEEEVIDENGEIVKRKKKKGLKEKIKEKLPGHKDHVEGEHVAAAPAPFVTHGGHHDDPAPVVTHGGHHDTDVVVEKFEGDVKTEAPPAPEEEKKGFLEKIKEKLPGGHKKPEDAAAVPATHAAPAPAHAPAPVTEEVSSPDGKEKKGILGKIMDKLPGYHGKAAGEEDKAATAAAAEHKTSG
Theobroma cacao	TcDHN-SK2	9.63	100-112	173	MEGHHAEGDHNKAVETTDRGMFDFLKKEDEDVAMADAHKPNNAKEKEKPKEKLHRADSNSSSSSDEEEEGEDGEKKKKKKGLKGKIKEKISGDKEATEHKDTSIPTANPEEKKGFLDKIKEKLPGQNKKPEDNALDGHPAEREPKEKGILEKIKEKIPGYHGHKTEEDKGKEN
Theobroma cacao	TcDHN-SK3	ND		234	MAEEHHSKAPECESNVAGEGAVETKDRGLFDFLGKKEEEKKPQEEVVIATEFDEKVKIEETKVEEEHKKEAGEEKKHSLLEKLHRSDSSSSSSSDEEEGEGEEKKKKKKEKKEKKGLKEKIEEKLGGEKKEEEMKHEDTSVPVEKCAEPVVQAEPEKKGFLEKIKEKLPGQYKKAEEVPPPPPPPPAECVAAEPHEGAEAKEKKGILEKIKEKLPGYHSKTEEEKEKEKESASH
Triticum aestivum	TaWCOR410-SK3	5.56<br>7.38	211-226<br>164-176	262	MEDERSTQSYQGGEAAEQVEVTDRGLLGNLLGKKKAEEDKEKEEELVTGMEKVSVEEPEVKKEEHEDGEKKETLFSKLHRSSSSSSSSSDEEEEEVIDDNGEVIKRKKKKGLKEKLQGKLPGHKDTEGEHVTGLPAPAAPASVQTHGGHHDTDVVVEKIDGDVKTEAAPAVPEEEKKGFLEKIKEKLPGGHKKPEDAAAVPVTHAAPAPVHAPVPAPEEVSSPDAKEKKGLLGKIMDKLP GYHKTGEEDKAAAATGEHKPSA
Triticum aestivum	TaWCOR410b-SK3	2.63<br>12.28	217-232<br>162-174	268	MEDERSTQSYQGGEAAEQVEVTDRGLLGNLLGKKKAEEDKEKKEEELVTGMEKVSVEEPEVKKEEHVDGEKKETLFSKLHRSSSSSSSSSDEEEEEVIDDNGEVIKRKKKKGLKEKLQEKLPGHKDTEGEHVTALPAPAAPASVQTHHDTDVVVEKIDGDVKTEATPAVPEEEKKGFLEKIKEKLPGGHKKPEDAAAVPVTHAAPAPVTHAAPAPVHAPAPAAEEVSSPDAKEKKGLLGKIMDKLPGYHKTGEEDKAAAATGEHKPSA
Triticum aestivum	TaWCOR410c-SK3	2.63<br>7.38	208-223<br>161-173	259	MEDERSTQSYQGGEAAEQVEVTDRGLLGNLLGKKKAEEDKEKKEEELVTGMEKVSVEEPEVKKEEHEDGEKKETLFSKLHRSSSSSSSSSDEEEEEVIDDNGEVIKRKKKKGLKEKLKEKLPGHKDTEGEHVTGLPAPAAPASVQTHHDTDVVVEKIDGDVKTEAAPAVPEEEKKGFLEKIKEKLPGGHKKPEDAAAVPVTHAAPAPVHAPVHAPAPAAEEVSSPDAKEKKGLLGKIMDKLPGYHKTGEEDKAAAAAGEHKPSA
Zea mays	ZmDHN-SK	14.01	193-205	289	MEDERNTQQHQGGEQAQDQENEVKDRGLLDSLLGRNKHDDQEKKNQQEEEELATGMEKVTVAEPDHKEEGHEAAEKKDSLLAKLHRTSSSSSSSSDEEEEVIDENGEIVKRKKKKGLKEKIKEKLPGHKDAEGDHHQPGVPAPAPAPPVAVDTHAHHQEGEHKPHFPAPAPPPHVETHHPVVVHKIEDDDTKTQTPPQAPEEEKKGLLDKIKEKLPGGHKKPEDAAAAAAPAVHAPPPPAPHAEVDVSSPDGKKGLLGKIMEKLPGYHKGSGEEDRKDAAGEHKTSS
Zea mays	ZmDHN-SK3	0.49<br>1.19	235-252<br>139-153	290	MEDERNTQQHQGGEAQQDAADQVDVKDRGLLDSLLGRKKHDDDQEKKKQTDELATGMEKVTVSEPEKHGHKEEEHEVVGEKKEGLLAKLHRTSSSSSSSSDEEEVIDENGEIIKRKKKKMGLKEKLKEKLPGHKDGHHTAAPSPAPAPVETHAHHQEEAHRPHVVPTPAPPPPPPHVHQHDHGVVVQDDVKTGTPPHAPEEEKKGLLDKIKEKLPGGHNKKPEAAAAAAPAPPVHAPAPAPQAENVSSPDGKEKKGLLGKIMDKLPGYHKSSAEEADHKADAAGEHKTTSS

YSK-Type

Specie	Access number/Name	PEST score	Position	Protein size	Sequence
Arabidopsis thaliana	AT3G50980-YSK2(Xero/<br>LT145)	ND		128	MESYQNQSGAQQTHQQLDQFGNPFPATTGAYGTAGGAPAVAEGGGLSGMLHRSGSSSSSSSEDDGLGGRRRKKKGITEKIKEKLPGHHDSNKTSSLGSTTTAYDTGTVHHEKKGMMEKIKEKLPGGHH
Arabidopsis thaliana	AT5G66400-Y2SK2(RAB<br>18)	ND		186	MASYQNRPGGQATDEYGNPIQQQYDEYGNPMGGGGYGTGGGGGATGGQGYGTGGQGYGSGGGQGYGTGGQGYGTGTGTEGFGTGGGARHHGQEQLHKESGGGLGGMLHRSGSGSSSSSEDDGQGGRRKKGITQKIKEKLPGHHDQSGQAQAMGGMGSGYDAGGYGGEHHEKKGMMDKIKEKLPGGGR
Avicennia marina	AmDHN1-YSK2	2.92	61-91	195	MAEYGDQYGRQTDEYGNPIRQTGEFGATGAYGANQQYGTTGTTVGYGTDQCVTTVTTGAQKTDQYGTPGTTGAYGTDQYGTTGTTGEYGTHGGGIAPGATDAGLAGGGGGHRRLGSGSSSSSEDDGQGGRRKKGIKEKIKEKLPGGGHGDDQTHPTPPGSGGYGYEHGGAAESPEHEEKKGIMDKIKEKLPGGHH
Brachypodium distachyon	BdDHN-YSK	ND		157	MDRYQGQQGYGANQVDEYGNPVARHGATGGMGGTYEAPGAFTGTGGGQGGGQFQPTNGEHKTGGILHRSGSSSSSSSEDDGVGGRRKKGMKEKIKEKFPGGHKENQQHMAAGTGGAYGQQGGYGGMTGTGAGAHGVDTGEKKGLMDKIKEKLPGQH
Coffea canephora	CcaDHN-Y3SK	ND		162	MADLRDEYGNPMQLTDQYGNPVQLKDEYGNPMQLSGVAITAGTASAVHSTGTGPTAATGTGQHQEQLHRSSSSSSGSSEDDGQGGRRKKKGLKEKIKEKLTGGRHKDRDDQEHIDDQHAHSASPPTTTTGSGTSTTVGGQQHEKKSMVEKIMEKLPGHHDTR
Coffea canephora	CcaDHN-Y2SK2	ND		172	MAQYGAEYGNQKSQYDEYGNPVRQTDEYGNPARHGGTMGDYGTTGTTGAYGGTTGAHGTYATGTTGTTGTGAYATQPGTDVGKEHHGLGGMLHRSGSGSSSSSEDDGQGGRRKKGMKEKIKEKLPGGHKEAQPGQEYSSATAAPGYGGEGVQHEKKGIMDKIKEKLPGGHHN
Hordeum vulgare	DHN1-SK	ND		139	MEYQGQHGHATDKVEEYGQPVAGHGGFTGGPTGTHGAAGVGGAQLQATRDGHKTDGVLRRSGSSSSSSSEDDGVGGRRKKGMKEKIKEKLPGGAHKDAAGQQQQTAMAGEYAGTHGTEATGEKKGVMDKIKEKLPGGQH
Hordeum vulgare	DHN2-SK	ND		143	MEYQGQTGHATTDKVEDYGQPVAGHGGATGGPTGTHGAAAAAAGTGQLQPTRDDHKTDGVLRRSGSSSSSSSEDDGVGGRRKKGMKEKIKEKLPGGAHKDAAGQQHTPAAGEYAGTGTHGAEATGEKKGVMDKIKEKLPG GQH
Hordeum vulgare	DHN3-YSK	ND		155	MEHGHATNRVDEYGNPVAGHGVGTGMGTGAAAGGHFQPTREEHKAGGILQRSGSSSSSSSEDDGMGGRRKKGLKDKIKEKLPGGHGDQQQTGGTYGQHGHTGMTGTGEHGATATGGTYGQQGHTGMTGTGAHSTDGTGEK KGIMDKIKEKLPGQH
Hordeum vulgare	DHN4-YSK	ND		247	MEYQGQQQQHGRVDEYGNPVAGHGVGTGMGTHGGVGTGAAAGGHYQPMRDEHQTGRGILHRSGSSSSSSSEDDGMGGRRKKGIKEKIKEKLPGGHGDQQHNAGTYGYGQQGTGMAGTGGTYGQQGHTGMTGMGATDGTYGQQGHTGMAGTGAHGTAATGGTYGQQGHTGMTGTGMHGTGGTYGQQGHTGMTGTGMHGTGGTYGQQGHTGMTGTGMXGTGGTYGQHGTDTGEKKGIMDKIKEKLPGQH
Hordeum vulgare	DHN6-YSK	ND		502	MAHFQGQQHGHPATRVDEYGNPVTAGGHGGGVTGTDGLGHFQGQQHGRTTTRLDEYGNPVTAGHGVGAGSTGTEVHGPGHAGYGSTGTNDTGGHGRQVGYGATGTGTHDAGGYGGSGIAPRHGGAGTGVHDAGGLGHTTGHGATSTHGTGHTAGYGSTGTGMTGTHGTGHTAGYDATGTGITGTHGTGHTTGYGGTGTHGTGHTAGYDATGTGIAGTHGTGRTAGYGGTGTGTHGTGHTTGYGGGATGTGTHGTGHTAGLGGTGTGTGITGTHGTGHTTGYGGGVTGTGITGTHGTGHTTGLGGTGTGTGTHGTGHTAGLGGTGTGTHDVVGTHPHGGLGEHKTGGILHRSGSSSSSSSEDDGMGGRRKKGMKQKIKEKLPGGNKEQTTATGGYGPGYTGTG
Hordeum vulgare	DHN7-YSK	ND		181	MEYQGQQQHGQATNRVDEYGNPVAGHGGGTGMGAHGGVGTGAAAGGHFQPTRKEHKAGGILQRSGSSSSSSSEDDGMGGRRKKGIKDKIKEKLPGGHGDQQHAAGTYGQQGIGMAGTGGTYGQQGHTGMTGTGATGTMAT GGTYGQQGHTGMTGTGTHGTDTGEKKGIMDKIKEKLPGQH
Hordeum vulgare	DHN9-YSK	ND		146	MEFQGQHDNPANRVDEYGNPFPLAGGMGGAHAAPGTGGQFQARREEHKTRGILHRSGSSSSSSSSEDDGMGGRRKKGMKEKIKEKLPGGHKDNQQHMATGTGTGGAYGPGTGTGGAYGQQGHAGMAGTGEKKGIMDKIKE KLPGQH
Hordeum vulgare	DHN11-YSK	ND		232	MADYGGEYGHPYPRVDEYGNPVPPVDQYGNPIPREPGQAPAYGSAGAVTPAAEYGAGVKPGYGLGGAVHPHESVVGGAVPPSGAAYAHEGAVSGGLAPGETTAYAYEGAVSGGLAPGETTAYAYEGMVGSGIGAGAGDRIQPAKEEHTTLGEALRRSGSSSSSSSSSSEDDGQGGRQRKKKGIKEKIKEKLPGNQKHEEHKAGHAAAPAAGTGTHEKKGIMEKIKEKLPGHH
Lycopersicum	LyDHN-Y2SK	ND		157	MAHYENQYSAGQALQTDEYGNPVRQTDEYGNPIQQTGGTMGEYGTTGTGYGTGQAGHTSGVLGGEQRQHGTLGGMLHRSGSSSSSSSEDDGHGGRRKKGIKDKVKEKLPGGHRDDQAHSTATTTTGYGMEGTHEKKGIMEKIKEKLPGHHGPGHH
Malus domestica	MdDHN1-YSK3	3.61<br>10.19	102-122<br>152-169	188	MSHLHNQHGAQPTDAYGNPIQRTDEYGNPLGGATTGAATGGYGAHDTAAATTGHDYGRKEHHGVTGALHRSGSSSSSSSEDDGLGGRRRKKGLKQKTKEKLHGSTTTDTTYGTAGTTYPGGHQQEKGMMDKIKDKLPGTGHKDHPHYSTTTHATTTTTPSGGATYTEEHHEKKGIMDKIKEKLPGGNH
Malus domestica	MdDHN2-Y2SK3	0.57<br>0.57	106-127<br>142-163	230	MSNYGNTTAEKTTEEYFGNPTYNGSAGADRTDEFGNAVQHGRTNTGYGTADPKTRDEGIVGHHGGPAVLQRSGSSSSSSSEDDGLGGRRKKGLKEKIKEKLPGGGNKDDQYSGGTQTTTPYGGGTTYKVGEQRQEKLPGGGNKDDQYSGGTQTTTPYGGGTTYKVGEQHQEKGTTDKIKKKLPGGNRDDQYSHDTSTTAAYGGTGRTGEPQEKKNMMDKIKEKLPGGHAE
Malus domestica	MdDHN4-YSK3	0.72	109-129	232	MANYGSTPEKTNENYGNPTYNGSAGAGRTDEGLNVVQHGRTDTGYGTAHAKPGDEVIVGRHGGPAVLQRSGSSSSSEDDGLGGRRKKGLKEKIKEKLPGGGNKDDQYTHGTQTTAPYGGGTTYTVGEGEHHQKRGVMDKVKEKLPGGGNKDDQYSHGTQTATPYGGGTTYAGEGERDQKKGVMDKVKEQLPGGASKDDQYSHAKPWDHLCRRRRAPPEKGCDGQGKGEAARRC
Oryza sativa	OsRAB16B-YSK2	ND		164	MDNYQGQHGYGADRVDVYGNPVGAGQYGGGATAPGGGHGAMGMGGHAGAGAGGQFQPAREDHKTGGILHRSGSSSSSSSEDDGMGGRRKKGIKEKIKEKLPGGNKGNNHQQQMMGNTGGAYGQQGHAGMTGAGTGVHGAEYGNAGEKKGFMDKIKEKLPGQH
Oryza sativa	OsRAB16C-YSK2	ND		164	MENYQGQHGYGADRVDVYGNPVAGQYGGGATAPGGGHGVMGMGGHHAGAGGQFQPVKEEHKTGGILHRSGSSSSSSSEDDGMGGRRKKGIKEKIKEKLPGGNKGNNQQQQQMMGNTGGAYGQQGHAGMTGAGTGTGVHGAEYGNTGEKKGFMDKIKEKLPGQH
Oryza sativa	OsRAB16D-YSK2	ND		151	MEYQGQHGGHASSRADEHGNPAVTTGNAPTGMGAGHIQEPAREDKKTDGVLRRSGSSSSSSSEDDGMGGRRKKGIKEKIKEKLPGGNKGNNQQQQQEHTTTTTGGAYGPQGHDTKIATGAHGGTAATTADAGGEKKGIVDKIKEKLPGQH
Oryza sativa	OsRAB21-YSK2	ND		172	MEHQGQHGHVTSRVDEYGNPVGTGAGHGQMGTAGMGTHGTTGGMGTHGTTGGMGTHGTTGTGGGQFQPMREEHKTGGVLQRSGSSSSSSSEDDGMGGRRKKGIKEKIKEKLPGGNKGEQQHAMGGTGGAYGQQGHGTGMTTGTTGAHGTTTTDTGEKKGIMDKIKEKLPGQH
Oryza sativa	OsRAB25-YSK	ND		228	MAEHATGVYGHPYPRVDQYGNPVPPVDQYGNPVPDEPAPRDTAAGYVAPPDPAVSTGDYGLAGAEAPAPARERGDERRSRRCRRTRRRGVHARRRRRSSPGRREDVRLRGHGQRRRLTGASGQLQPTTREEGHTTLGETLRRSGKSSSSSSSSEDDGQGGRRKKKSIKEKIKEKLPGSHKQEEQKQAGHTAPAAGTGTGTGTHAAGKHEKKGIVEKIKEKLPGHGHH
Populus trichocarpa	PtrDHN-2-Y3SK2	5.32	149-165	183	MIAATIVDELGNPIQLTDEHGNPVKLTDEHGNPVHIAGVATTKQPPTLGDIISTDTVPGTGHLSSTARSEDAMKGGLRETGHHGEVAGDQGGHKKEEHDETSTSSTSSSGSSEDDGQGGRRKKKKGLKQKIKEKLTGGKHKEEHGHTVDVHTTTTGPAGEQYQEQEKKSVIEKIKGKLPGHHSHH
Prunus pérsica	PpeDHN3-Y2SK3	2.88<br>4.88	33-52<br>167-184	202	MASYEKQYGATPTDEYGNPIRRTDEYGNPLGHHTTTGTTAATTGAGGYDPHDFAATTVAGQGGQVHGHDYDRKGHHGVTGAVLHRSGSSSSSSSEDDGLGGRRKKGLKEKVKEKLPGGTKTDTTYGGTGTTGQHHQEKGTMDKIKEKLPGTGGHRADDPYPSQTHTTATTPYGGTAYTEEHQEKKGIMDKIKEKLPGGHH
Setaria itálica	SitDHN1-Y2SK2	ND		223	MAEYHGEYGHPYPRVDQYGNPVPPVDQYGNPVPREPASAGLGGTAPLYGAAGDTTGAGYVAAPPGDYGTAGGAAYPSSGVAPGETALAYEGMVGGGGIGAATAGMGGQLQPTREEEHTFGSSQLEPASREEHTTLGEKLTRSGSSSSSSSSFSEDDGQGGRRKKKSIKEKIKEKLPGTHKHEERKAGHAAPATTTTGTTHAAGAHEKKGFMDKIKEKLPGHH
Setaria itálica	SitDHN4-YSK2	ND		169	MEYGQQGQHGHGTAGRVDQYGNPVGGVGHGTTGTGTGGMGTGYGTTGTGGMGQLGEHGGAGMGGGQFQPVREEHNTGGILHRSGSSSSSSSEDDGMGGRRKKGIKEKIKEKLPGGRKDDQHATATGGTYGQQGHTGMTGTGTGGAYGTEGTGEKKGIMDKIKEKLPGQH
Solanum lycopersicum	SlTAS14-YSK2	ND		130	MAQYGNQDQMRKTDEYGNHVQETGVYQGTGTGGMMGGTGTGGMMGGTGGEYGTGQMGTGTHHHEGQQQLRRSDSSSSSEDDGEGGRRKKGLKEKIMEKMPGQHEGEYGQTTGEEKKGMMDKIKDKIPGMH
Sorghum bicolor	SbiDHN-YSK2	ND		152	MEYGQQGQHGHGTTGRVDQYGNPVGAGTGGMGTGTGGMGQLGEHGGAGMGGGQFQPAREEHKTGGILHRSGSSSSSSSEDDGMGGRRKKGIKEKIKEKLPGGHKDNQHATATGGAAYGQQEHTGAGTYGTEGTGEKKGIMDKIKEKLPGQH
Theobroma cacao	TcDHN-YSK	ND		169	MADLRDVHGNPIQLTDEQGNPVQPTDEYGNPVHVTGIASKHPVMTQTGVPGGQMGYETGSAAGYQQQQHPQQLHYEASSGVEIQRSNSSSSGSSEDNEIGGRRNKKGLKDKIKDKLTGGKHKEEAQSQTTTYESKTTTTGVSHGQHHTEHEKKTMMEKIKEKLPGCHSH
Triticum aestivum	TaDHN7-Y2SK2	ND		213	MADYGGEYGHPYPRVDEYGNPVPPVDQYGNPIPREPGQVPAYTSGGAAPPYSSDGAGAVTSADYGAGVTPGYGLSGAVHPQESVVGGAVFPSGTAHTHEGALSGSLAPGETTAYAYEGMVGSGIGTGDQIQPTKEGHTTLGETLRRSSSSSSSSSSEDDGQGGRQRKKKSMKAKIKEKLPGSHKQEEHKAGHTVPPAGTGTHEKIKEKLPGHH
Triticum aestivum	TaWZY2-12-YSK2	ND		152	MEYQGHQQHGQATNRVDEYGNPVAGHGVGTGAAAGGHFQPSGEEHKAGGILQRSGSSSSSSSEDDGMGGRRKKGIKDKIKEKLPGGHGDQQQTTDNTYGQQGHTAGMAGTGGTYGQPGHTGMAGTGTHGTDGTGEKKGV MDKIKEKLPGQH
Triticum aestivum	TaWZY1-1-15YSK2	ND		160	MEHGQATIRVDEYGNPVAGHGVGTGMGAHGGSGTGAATGGHFQPTREEHKAGGILQRSGSSSSSSSEDDGMGGRRKKGIKDKIKEKLPGGHGDQQHADGTYGQQGTGMAGTGAHGSAATGGTYGQPGHTGMTGTGTHVTD GAGEKKGIMDKIKEKLPGQH
Triticum turgidum	TtDHN15.1-YSK2	ND		149	MEFQGQHDNPANRVDEYGNPFPLGGVGGAHAAPGTGGQLQARRGEHKTGGILHRSGSSSSSSSSSEDDGMGGRRKKGMKEKIKEKLPGGHKDNQQHMATGTGTGGAYGPGTGTGGAYGQQGHAGMAGAGTGTGEKKGIMDKIKEKLPGQH
Triticum turgidum	TtDHN11-YSK2	ND		158	MEYQGQQQHGQAATNRFAEYGNLVAGHGAGTGMAAHGGVGTGAVAAAGGHFQPTREEHKAGGILQRSGSSSSSSSEDDGMGGRRKKGIKDKIKEKLPGGHGDQQQTAGTYGQQGHTGTAGTGGTYGQPGHTGMAGTDSTGEKKGIMDKIKEKLPGQH
Triticum turgidum	TtDHN18-YSK2	ND		227	MEFQGQHDNPANRVDEYGNPFPLAGGVGGAHAAPGTGGQFQAHRGEHKTGGILHRSGSSSSSSSSEDDGMGGRRKKGMKEKIKEKLPGGHGDQQHTGGTYRQQGTGMVGTGGTYGQKGHTGMTGTGGTYGQQGHTGMTGTGGTYGQQGHTGMTGTGGTYGQQGHTGMAGTGAHGTTATGGTYGQPGHTGMTGTGAHGTGGTYGQHGTDTG EKKGIMDKIKEKLPGQH
Utricularia Gibba	UgDHN-YSK	1.64<br>7.24	87-102<br>102-115	133	MAELRDEHGNPVQLTDQFGNPVQLTDEYGNPVHLKGVATPEHAREQLQRSGSSSSSSSEDDGQGGRRKKGLKEKVKEAIGGGKHQEKEIAPETAMTTMAEEKTPTDAGGAPEQEKKGIMEKIKEKLPGHHRQH
Vitis riparia	VrDHN1-YSK	ND		130	MAYQQDPCANPTRQTGKTGGQTDQYGNPVHQTEALGAYGAGTGTGMHGGEHQQQPHQQPGVLHRSGSSSSEDDGQGGRRKKGMKEKIKERIPGMGRKDEQKQTSATSTPGQGQQQKGMMEKIKEKLPGAH
Vitis riparia	VrDHN1a-YSK2	ND		130	MAYQQDPCANPTRQTGKTGGQTDQYGNPVHQTEALGAYGAGTGTGMHGGEHQQQPHQQPGVLNRSGSSSSEDDGQGGRRKKGMKEKIKERIPGMGRKDEQKQTSATSTPGQGQQQKGMMEKIKEKLPGAH
Vitis vinifera	VvDHN1a-YSK	ND		130	MAYQQDPCANPTHQTGKTGGQTDQYGNPVHQTEAMGAYGAGTGTGMHGGEHQQQPHQQPGVLHRSGSSSSEDDGQGGRRKKGMKEKIKERIPGMGRKDEQKHTSATSTPGQGQQQKGMMEKIKEKLPGGH
Zea mays	ZmRAB17-YSK2	ND		168	MEYGQQGQRGHGATGHVDQYGNPVGGVEHGTGGMRHGTGTTGGMGQLGEHGGAGMGGGQFQPAREEHKTGGILHRSGSSSSSSSEDDGMGGRRKKGIKEKIKEKLPGGHKDDQHATATTGGAYGQQGHTGSAYGQQGHTG GAYATGTEGTGEKKGIMDKIKEKLPGQH
Zea mays	Zm00008a019720	1.76	22-44	169	MTVDASVTNGDFFTYPFVLNKHFLAEVSGDSDDGLDEGSLTAPKRANQDHRRIAFSSLPFPEQREVATSMSGIIDKIQETLHGGDHKEEHEKHKGEEHHKKDDGEHKEGIVEKIKDKITGEHGDKSGDHKDKDHKEKKDKKKKKEKKHGEGHDHGDGDGHSSSSSDSD

K-Type

Specie	Access number/Name	PEST score	Position	Protein size	Sequence
Aquilegia coerulea	AcoDHN-K	ND		163	MATEQNQIPKAHVTNEEEGQHEHEHHTMLDFLEKKEEQKPTDELSVNEFVKDHVSKSEEKKEEEEHKPTSAPNVDRSSDIHSSGDVTHPEETEEKNLMEKIKNKLPGAHKKTDEVAPVEHHPTETKTNIHQDKPEEKKGLMEKIKEKLPGHNKSVEEEKGAI
Arabidopsis halleri	Araha.12526s0002-K2	ND		106	MQSSDEEAGENGEKKEKEKKNKKKKEVVADQCETEEKNKEKLPAGIGHEDGKEKGFMEKMKDKLPGGHHGKPEAEPHNDKGKEKGFMEKIKEKLPGHTNDEKKKET
Arabidopsis halleri	Araha.41700s0001-K2	ND		71	MHEGGGWTRWNGRRRKKKGITEKIKEKLSGHHESNKTSLGSTTTAYDTGTVHHEKKGMMEKIKEKLPGGHY
Arabidopsis lyrata	XP_020882347.1<br>dehydrin Xero 2	ND		213	MDSYQNQTGVPKKGMTEKIMEKLPGHHGTHQTHGTNTGYGNTGGVHHEKKGMTEKVMEQLPGHHGTHQTHGTNTGYGNTGGVHHERKGMTEKVMEQLPGHHGTHQTHGTNTGYGNTGGVHHEKKSMTEKVMEKLPGHQGSHQTGTNTGYGATNTNVVHHQKKGIAEKIKEQLPGHHGAHQTGTTANYGNTGVVHHEKKSTIDKIKEKLPGGHH
Arabidopsis thaliana	AT3G50970-K6(Xero2/<br>LT130)	ND		193	MNSHQNQTGVQKKGITEKIMEKLPGHHGPTNTGVVHHEKKGMTEKVMEQLPGHHGATGTGGVHHEKKGMTEKVMEQLPGHHGSHQTGTNTTYGTTNTGGVHHEKKSVTEKVMEKLPGHHGSHQTGTNTAYGTNTNVVHHEKKGIAEKIKEQLPGHHGTHKTGTTTSYGNTGVVHHENKSTMDKIKEKLPGGHH
Boechera stricta	Bostr.6864s0147-K6	ND		216	MNSYQNQSGVQKKGITEKIMEKLPGHHGSHQTGVNTAHGASGTGGVHQGPTEKIMEQLPGHHGSHQTGTNTAYGNTGGVHHERKGMTEKIMEKLPGHQGSHQTGTNTAHGATNTNVVHYEKKGIAEKIKEQLPGHHATHHTGTTTDHNTTTGHNTTGTGVVHHEKKGIAEKIKEQLPGHHGSHQTGTTTGYGNTGVVHHENKSTMDKIKEKLPGSH
Capsella grandiflora	Cagra.2007s0059-K2	ND		143	MASYQNRPGGATGGTYGTGGQGYGTGTGTEAFGTGGGARHHGQEQLHKESGGGLGGMLHRSGSGSSSSSEDDGQGGRRKKGITQKIKEKLPGHHDQSSQAQGMGGMGTGTTGYDAGGYGGERHEKKGMMDKIKEKLPGGGGR
Citrus clementina	*Ciclev10029952m.g-K4*	8.52	42-58	167	MADHQQQGNEAKGCGMFDFLKKNKEEKSQDAAMVDVETKEEKPAFSSEEEIEGADGEKKRKKKKGLKEKIQEKISSEKEENAVEKNDEIASAEATHLEEKKGFLEKIKDKLPGQHKKAEEGAACGCCPNEHLAEGDEKEKKGILEKIKEKLPGYHSEDEKAKHKDN
Citrus sinensis	orange1.1g036567m.g-<br>K	ND		73	MSGVIHEIGEALHVGGGQKEDKHKGEHHSGDQHTTDVHHQQQYHGGEHREGEHKEGLVDKIKQKIPGARRRPR
Glycine max	Glyma.08G048900-K	ND		91	MAHYQNQYGAVPVSKDPIQKEKETNGDTTGIAVDGGGVAGSTQVIAEEDYDHRRTTTTTTTTTAVDNHPHKKGIIEKIKEKLPGTHHHNNK
Gossypium raimondii	Gorai.013G180700-K	ND		117	MLPMFRLHTLAGSFSCSLLVQHILFLQKNINKDASMELLDFSQGFSGTHFKGKSSSFQKKLGFLKSGKEGSKNLQNDKKQKGETKEKKRFLEKIKEKIPGYHSKTEDEKEKETTAPH
Hordeum vulgare	DHN5-K	ND		575	MEHQAHVAGEKKGIMEKIKEKLPGGHGDHKQAADTHGQQGHAGTGTHGAPATGGAYEQQGRTGVTGTGLNGADAGEKKGLMEKIKDKLPGGHGDQQTTAGTYGQQGHTGTAAHGTPAGGGTYDQEGHTGMSGMGAHDTHTTGGAYGQHAHTGGTGTGTHGTGEKKGVMENIKDKLPGGHGDHQKTSDTYGHQEDTEMTGMGRHSTPATGGSYGQHVHTGQTDMGTHGTGEKKGVMENIKDKLPGGHDDHQKTAGTYGQQGHVGTGTHGTPATGGAYGQHEHTGATGTGTYGTGEKKGIMENIKEKLPGGHGGHQKTGDTYGHQEDTKMTGMGRHSAPATGGSYGQHAHTGETGTGTHGTGEKKGVMENIKEKLPGGHDDHQKTAGTYGQQGHVGTGTHGTPATGGAYG
Malus domestica	MdDHN3-K4	ND		177	MDKVKEQLPGGASKDDQYSHAKPTTGAHYGGGTTYAGEGEHHQKKGVTDKVKEKLPGGASKDDQYLHAKPTTGAQYGGGTTYAGEGEHHQEKGVMDKIKDKLPFGGSKDGEGERHQEKGMMDKTKEKLPGGHKDNQYSHDTSTTAATYGGTANIGEHQEKKGIMEKVKEKLPGGHKK
Malus domestica	MdDHN7-K3	7.69	38-56	124	MAGKSTMVPLACFIALAAQARALEVVGGEGLKQKIKEKLPGSTTTDTTYDTTYPGRHHQEKGMKDKNKDKLPEGHKDDPYYSTPHTTLTTTTYGVTTYMEEHHEKKGIMDKINEKLPGSHHWRL
Malus domestica	MDP0000689622-K2	ND		83	MDKIKDKLPFGGSKDGEGERHQEKGMMDKTKEKLPGGHKDNQYSHDTSTTAATYGGTANIGEHQEKKGIMEKVKEKLPGGHKK
Malus domestica	MDP0000265874-K3	5.22<br>7.78<br>0.84	11-55<br>98-133<br>156-171	190	MANYQSQYGTKPQDTDEXGNPIQQGTTGGFGSATTGTGYGTTETPYTGGQQQQERGTGMTGSGYGTTDAPYTGGQQQQERGMMDKIKDKIPGTGGGRRDDDPYSSNAQTGTGMTGTGYGTTDAPYTGGEYEERGMMDKVKDKIPGTGQRDETYSSKTASTTAPYGGTTGEHQEKKGAMDKIKEKLPGGRH
Malus domestica	MDP0000178973-K5	ND		210	MADQYPKESQTQSKGAEEEAQGCGIFDFLKKKDSEEKPHEEQEQQKHTTIAEKLHHHGGGENKKKGLKGKMKEKVSGNKEEGDAYHASVPAKNGHSDEKGFVEKIKDKLPGQHKEAEHXAEEKGFVEKIKDKLPGQHKVAEHGAREKGFVEKIKEKLPGQHKEAGNGAHAEYHAPDGHSHDSEPKKGVLEKIKDKLPGGHKNEEKKHKEY
Malus domestica	MDP0000595271-K3	3.21<br>4.67	29-51<br>81-98	117	MVALLDQSEDDGLGGRRKKKGLKQKIKEKLPGSTTTETTYGTAGTTYPGGHQQEKGMMDKIKDKLPGTGHKDDPHYSTTTHASTTTTPYGGATYTEEHHEKKGIMDKIKEKLPGGNQ
Medicago truncatula	Medtr8g106140-K	ND		85	MDNFQNQYGAVPISNNDLIHRELDTTGVVDGGGSTQAMADTTTAVDTTTTDDGVFVDGNKTNNHHNKKGIIDKIKEKLPGTHHHK
Oryza sativa	OsLIP9-K2	7.14<br>14.84	60-85<br>85-99	137	MATPAPVVTHGDHHHDTAVPVEKIEGDHAKTEATLPRAPEEEKKGFLDKIKEKLPGGHKKPEDATAVPPPAASPAAPATTPAPAHPPPATEEVSSPDGKEKKGILGKIMEKLPGYHKGSGEEDKTAAAATGEHKSSA
Phaseolus vulgaris	Phvul.002G324800-K	ND		82	MAQYQNQYGAVPISKDPVQKEMETTGDTIGVGVAGGGGGGSTQVIAEDYDSSKSNSSGEKQKKGIIEKIKEKLPGTGTHQDK
Physcomitrella patens	PpDHNB-K	8.96<br>6.26<br>7.32<br>9.39<br>3.78	22-53<br>171-196<br>197-221<br>102-118<br>279-290	290	MAAQYTQDQSTEFRPELDEPRRTTTTTTSTTGSGLENENFGGYGGVSENEPPRHKIHSEDEPLPKPMSGTYLDEEGAKLDQDSDRSGLGASDLGRDEHIMPKPTSEGYPAGTPQSTEKYQEHRDLEPTRLDESPKTEEFGAANASTGDFDRTSSDGLRTDKTPASEPRGFRSEDTAPTSGGYADPTSAFPGAPIDRRVEEPGYGYDQQTSEPTSLESGTQHSPKKEGFMTKLKEKLPGHHKTPESGVEHQGAGVEHDTTDAPPKKGLMTKIKEKLPGHHSTAPASTTTDP
Physcomitrella patens	PpDHNC-K	4.89<br>8.35<br>4.70<br>2.84	121-149<br>158-176<br>63-79<br>44-58	233	MAGYMGEERKPVSTYNAHQDEFGKEGNDYPASGGGYGDHHHRPDVPSSTPGEYGGPRGQVEHGYGDQSEETFQDAPERLTGYGDTDNGVGGPKSGYGDSRESGTAYDRATDEQTQFGVGGKENSYELEGSDPQLGGTDTSSYAAVDPQRLDSDRSPIRATETVPGGDGWGPEDTSRIHGAKKDGLMNKIKEKLPGHHNTAQGEVSDPNALPKKGMMEKIKEKLPGHDSGSADV
Pistacia vera	Pv-dhn-K5	ND		230	MSGTINKVKETLHMGGQKKEGEQKGLYQGEAHKVEQHHGVCKPEHGEHKEGMVQKIKNKVPGGQDGVKQGHGVCKPEHGEHKEGLVEKIKDKVPGQGDQHGHGMQQHRGEY
Populus trichocarpa	PtrDHN-1.1	6.06<br>10.60<br>21.67<br>17.03<br>5.83<br>8.97	365-385<br>401-419<br>65-82<br>264-280<br>149-163<br>38-49	444	MAGVNKSHEYETKTSAGEESGATETKDRGLFGFMGKKKEEVPATEYEEKIHRSDNSYPGDGEKKHEDTTVPSDTETPLTPEKKKSYFEQAKGIIPAYKKTEEVPPLELKLFIRQGHPLPQRRRRRHPLTPEKKKSYFEQAKDIIPAYKKTEDGPPSPAEAAVHPTGTPLEPEKKKSYFEQAKDRYQHIRKLKSPLPVQLKPQFIPQEKKSFFEQAKERTPGFKKTEEVSPRPAKAAAHHTETPFEPEKKKSFFEQAKERIPSYKKTEEVISTETPLEPEEKKGFFDQAKERIPAHKTEEVPPHPAQSASNSFSHTETPFEPEEKKGFLDKVKEKDLARKKTEEKRGFLKEKVPTHKKTEEFPFPAKPAYTEAAVS[illegible]
Populus trichocarpa	PtrDHN-1.2	11.72<br>10.61<br>6.06<br>23.31<br>5.37<br>15.05	99-121<br>470-490<br>537-557<br>138-156<br>573-591<br>65-82	616	MAGVNKSHEYETKTSAGEESGATETKDRGLFGFMGKKKEEVPATEYEEKIHRSDNNYPGDGEKKHEDTTVPSNTETPLTQEKKKSYFEQAKDMIPAYKKIEEVPPPTEAVYPSETPLTPEKKKSYFEQAKDMIPAYKKTEEVPPPTESETPLTPEKKKSYFEQAKGIIPAYKKTEDGPPSPAEAAVHPTGTPLEPEKKKSYFEQAKDIIPAYKKTEDSPPSPAEAAVHPTGSRPLEPEKKKSYLEQAKDIIPAYKKTEDSPPSPAEAAAHPTGSRPLEPEKKKSYFEQAKDIIPAYKKTEDSPPSPAEAAAHPTGSRPLEPEKKKSYFEQAKERTPGYKKTEEVSPRPAEAVVHHTETPLEPEKKGFLDQAKERTPGY[illegible]
Populus trichocarpa	PtrDHN-4-K	ND		149	MAGIMHKIEETLNIGGKKDERKGETQGGYNQQDHRGAAQGQRKEGFADQMKDKIPGGGGGVHKGETQGGYNHQDQGGYNQQEHRGGAQGERQEGFVGQMKDKLPGGGGGGGMTQGGYNQQEHRGGAQGERQEGFVGQMKDKLPGGVAVA
Populus trichocarpa	PtrDHN-5-K	ND		205	MTQGGYNQQEHRGGAQGQRKEGFVGQMKDKIPGAGGGGGGGGGMTQGGYNQQEHRGGAQGQRKEGFVDKIKGKIPGGGGASGVGGMTQGGYNQQDHRGGAQGVRKEGFVDKIKGKIPGGGGASGVGGMTQGGYNQQDHRGGAQGVRKEGFVDKIKGKIPGGGGASGVGGMRRWEINKITGAARKVCARKGVEEVEVLSTSVADYH
Populus trichocarpa	Potri.013G062300-K4	ND		218	MSARVRHKVGTTNRITGAPHKVSRQEGFVGQMKDKLPGGGGGGGMTQGGYNQQEHRGGAQGGYNQKEHRGGAQGERQEGFVGQMKDKLPGGGGGMTQGGYNQQEHRGGAQGDRQEGFVGQMKDKLPGGGGGMTQGGYNQQEHRGGGGVSGMTQGGYNQQEHRGGGGVGVGGMTQGGYNQQEHRGDAQGERKEGFVDKIKGKLPGGGRASGVRGEGGEK
Populus trichocarpa	Potri.013G062200-K5	ND		236	MAGIVHKIEDTLNIGGKKDERKGETQGGYNQQDHRGGAQGQRKEGFVGQMQDKILGGGGGGGGMTQGGYNQQEHRGGAQGQRKEGFVGQMKDKIPGGGGGGVGGMTQGGYNQQEHRGGAQGQRKEGFVDKIKGKIPGGGASGVGGMTQGGYNQQDHRGGAQVVRKEGFVDKIKGKIPGGGGASGVGGMTQGGYNQQDHRGGAQGVRKEGFVDKIKGKIPGGGGASGVRGVGGEK
Populus trichocarpa	Potri.002G013200-K3	9.71<br>15.64<br>6.06<br>10.60<br>5.83<br>9.20	339-362<br>52-74<br>463-483<br>499-517<br>91-105<br>132-146	542	MAGVNKSHEYETKTSAGEESGATETKDQTPLTPEKKKSYFEQAKDMIPAYKKTEEVPPPTEAVYPSETPLTPEKKKSYFEQAKGIIPAYKKTEDGPPSPAEAAVHPTGTPLEPEKKKSYFEQAKDMIPAYKKTEDSPPSPAEAAVHPTGSRPLEPEKKKSYFEQAKDMIPAYKKTEDSPPSPAEAAVHPTGSRPLEPEKKKSYFEQAKDIIPAYKKTEDSPPSPTEAAAHPTGSRPLEPEKKKSYFEQAKERTPGFKKSEEVSPRPAKTPLEPEEKKGFFEQAKERTPGFKKTEEVSPRPAKAAAHLTETPLEPEEKKGFFDQAKERIPSHKKTEEVPPHPAQSASNEGAFSQTETPFEPEEKKGFLDKVKEKVP[illegible]
Prunus persica	ppa005514m.g-K3	ND		457	MAHFGSTPEPTKTDEYGNPVHHTTTGTGRTDEFGNPVQHGVADTGYGTGAGYGTHTKPGVVEHHGVPAVLHSKDDQYSRDTQTTTGGYGGDGYTGGEHQEKKGLLGQLQDKLPGGNKDGQYSHDTQTTTGAYGGAGYTGGEHHEKKGIIGQVKDKLPGGQKDDQYCRDTHPTTGAYGGAGYTGGEHDHEKKGIIGQVKDKLPGGQKDDQYCRDTHPTTGAYGGAGYTGGEHQEKKGIIGQVKDKLPGGQKDDQYSHDTHKTTGAYGGAGYTGDDTREKKGGQKDDHYSHDTHPTTGAYGGAGYTGDDTREKKGIIDQVKDKLPGGQKDDHYSHETHPTTGAYGGAGYTGDDTREKKG[illegible]
Sarracenia purpurea	SapurV1A.0016s0910-<br>K3	14.85<br>11.66<br>1.66<br>12.76<br>16.64<br>7.65	466-495<br>511-530<br>378-396<br>124-139<br>163-178<br>202-217	557	MAAESKSHEYETKTQAGEESGGGLFGFMGKKKEEKPYAATEYEEKIHRSDNSSPVDEVNKDEDIKPEEKKSFLEQIKGIMPAYNNTGDAPSSPAKTVTEKPLEPAEEKKGYLDQIRERMPAYKKAEDAPSSPTETVAEKPLEPAEEKKSYLDQIKERLPSYGKAEDAPSSPTETVTEKPLEPAEEKKGYLDQIKERMPAYKKAEDAPSSPAGTVTEKPLEPEEKKGYLDQIKERMPTYKKAEETPSPYKKTEDASPPPVEAAVNRTETPLEPEEKKGFFEQDREGIPSNRRIDDASPYPAKAAVYDTEAPLEPEEKKGFIDRAKERIPTPKKTEEVPPPPAESAHSGYAVRNTETPLEPGDNKGFIDQTKDKI[illegible]
Sarracenia purpurea	SapurV1A.2014s0030-<br>K3	ND		214	MHIKNHFKDLKLKKRKTMAGIMQKIEETFGGKKEEQRKGESQAGLGQQGHNTQGGYGQQGQQGYNAQGECKEGFVDNMKDKLPGGVGGVSGGVGGVGMHKGETQGGYGQQGHNTQGAYGQQGQQGYNAQGERKEGFVDNIKDKLPGGGGVGSGGMRKGETQGAYGQQGHNTQGGYGQQGHKAQGERKEGLVDKIKHKIPGCGGGGVDGGGERKK
Solanum lycopersicum	Solyc01g065820.1-K2	ND		78	MKNILGVSEDDGQGGRRKKKGLKEKIKEKFTGGKHKNEEPHHQAHGVGTRTTTTTTEHEKKSMMEKIKEKLPGHHNHH
Theobroma cacao	Thecc1EG006526-K	9.63	91-103	164	MEGHHAEGDHNKAVETTDRGMFDFLKEDEDDRNGILPRIIIQVVYELFIYFLQSSDEEEEGEDGEKKKKKKGLKGKIKEKISGDKEAREHKDTSIPTANPEEKKGFLDKIKEKLPGQNKKPEDNASDGHPAEREPKEIGILEKIKEKIPGYHGHKTEEDKGKEN
Triticum aestivum	TaWCS120-K5	ND		391	MENQAHIAGEKKGIMEKIKEKLPGGHGDHKETAGTHGHPGTATHGAPATGGAYGQQGHAGTTGTGLHGAHAGEKKGVMENIKDKLPGGHQDHQQTGGTYGQQGHTGTATHGTPATGGTYGQQGHTGTATHGTPATGGTYGEQGHTGVTGTGTHGTGEKKGVMENIKEKLPGGHGDHQQTGGTYGQQGHTGTATHGTPAGGGTYEQHGHTGMTGTGTHGTGEKKGVMENIKDKLPGGHGDHQQTGGTYGQQGHTGTATQGTPAGGGTYEQHGHTGMTGAGTHSTGEKKGVMENIKEKLPGGHSDHQQTGGAYGQQGHTGTATHGTPAGGGTYGQHGHAGVIGTETHGTTATGGTHGQHGHTGTTGTGTHGSDGIGEKKSLMDKIKDKLPGQH

YK-Type

Specie	Access number/Name	PEST score	Position	Protein size	Sequence
Ammopiptanthus mongolicus	ABH01188.1	ND		183	MAHIQSGYGDQDRKTIDEYGNPVRQTDQHGNPVNPTTGTGAGYGESGGTGYGGPTGDTAGYGSTGYGTTPGDKAGYGTTTGGDGTTTGYGSGGGTGHGTTTGGTTGTTEYGSGGGTTGTTGYGSGGGQHEHQHQHHGREKEEHGHEFASHGAPGGYEAASGEQQYEKKGIMNKIKEKLPGHRDV
Ammopiptanthus nanus	ADF43064.1	ND		181	MAHIQSGYGDQDRKTTDEYGNPVRQTDQHGNPVNPTTGTGAGYGESGGTGYGGTTGDTAGYGSTGYGTTPGDKAGYGTTTGGDGTTTGYGSGGGTGHGTTTGGTTGTTEYGSGGGTIGTTGYGSGGGQNQHQHHGREKEEHGHEFASHGAPGGYEAASGEQQYEKKGIMNKIKEKLPGHRDV
Arabidopsis halleri	Araha.12619s0007-YK	2.04<br>6.13	84-102<br>112-130	150	MADLKDERGNPIYLTDEHGKPAQLMDEFGNPMHLTGVATTVPHLKESSYTGPHPITAPVTTTNTPHHAQPISVSHDPLQDHDLRWFGTNLPEEDWEDGEGARSKTTEETKSKPSAATETGTGSGSIQEPHEKKGFFKKIKEKLSGHRNLM
Arabidopsis lyrata	327864-Y4K	2.04	84-102	161	MADLKDERGNPIYLTDEHGKPAQLMDEFGNAMHLKGVATTVPHLKESSYTGPHPITAPVTTTNTPHHAQPISVSHDPLQDHDLRWFGTNLPEEDWEDGEGARSKTTEETKSKLGVNNPSAATVTGTGTGTGSGSGSIQEPHEKKGFFKKIKEKLSGHRNLM
Arabidopsis thaliana	AT4G39130-Y2K	ND		151	MADLKDERGNPIYLTDEHGKPAQLMDEFGNAMHLKGVATTVPHLKESSYTGPHPITAPVTTTNTPHHAQPISVSHDPLQDHDLRWFGTNLPEEDWEDGEGARSKTTEETKSKLGVNNPSAATVTGTGTGTGSGSGSIQEPHEKKGFFKKIKEKLSGHRNLM
Capsella grandiflora	Cagra.0926s0062-YK6	ND		204	MDSYQNQSGVQKKGMTEKIMEKLPGHHAPHQTGASTGYGATTTGGVHHEKKGMTEKIMEKLPGHAPHQTGANTAYGATTTGGVHHENKGMTEKVMEQLPGHHGSHQTGYGNTGGVHHHEKKGMTEKIMEKLPGHQGTNTGYGGATNTGVVHHEKKGIAEKIKEQLPGHHGTGTTTSYGNTGGVVHHHEKKSTMDKIKEKLPGSH
Capsella rubella	Carubv10018039m.g-<br>YK6	ND		204	MDSYQNQSGVQKKGMTEKIMEKLPGHHAPRQTGASTGYGATTTGGVHHEKKGMTEKIMEKLPGHAPHQTGANTAYGATTTGGVHHENKGMTEKVMEQLPGHHGSHQTGYGNTGGVHHHEKKGMTEKIMEKLPGNQGTNTGYGGATNTGVVHHEKKGIAEKIKEQLPGHHGTGTTTSYGNTGGVVHHHEKKSTMDKIKEKLPGSH
Cicer pinnatifidum	AAN77521.1	ND		195	MSQYNQGQYVDQTRTVDEYGNPIVQVDQYGNPINQSGVGMTGEAGRTFENPGLTGHDNSKSHTTSYGTHTGSGGGTGDGYGYTYNTRGGGDGGTYNTRGGSGMTGEVGKIFGTTDDTGNHHGVDQTTTDYGTHNTGSYGTHTGGYGNTNTGSGYGNTKVGQEFGREEPRHHGGEQQHGEKKGIMDKIKEKLPGTGH
Cichorium intybus	ACF15449.1	ND		261	MAQYGGDQQYGREGRQTDEYGNPIRKTDEYGNPLHSTTGGTMGDYGTTGHHQGVGHATGVQELIQKPAAVTVPPVRGWVPNIGHAAGVTGTGYGTGHQGLGTGGTGTDYKTSGGHGTTGHHGIGTEGHYGTGADTESYQNQPSATPFGGGGIGTGTGTGAGYEGLGVAGGQKQPSATPFAGSDTGTGTGAGYDRHGGEGYEKKGMMEKIKEKLPGGHSTDEHQRVSTTTAGGVGGGGYGEGGQTHEKKGVMEKIKEKLPGHH
Galega orientalis	ADT80777.1	ND		280	MSQYNQGQYGQQTRKTDEYGNPVVQVDQYGNPIGGMTDTSTGHQQQHHGLDQGTGYGTHIGKGTGYGTHTDIITGTGYESHTGSHGTGYGTGTGTGGYGTHTGEYGSTNAGSGFGNTNLDGTGTGYGTTGYGGTGSTGIRHGDYQHGEEKGIMDKIKEKIPGTGNTNTGSGYGTTGYGTGYGTTGYGGTGSTGVRHGDHQHGEDKGIMDKIKEKIPGTGNTNTGSGYGTTGYGGTGSTGVGQEYVREERHHHGDQQQHGEKKGIMDKIKEKLPGTGSCTGH
Glycine max	Glyma.09G185500-Y2K	ND		253	MLACIFHHPTSHTTTSTSLQSKPTTTMASYQKHYDDQGRKVDEYGNVEKQTDEYGNPVHAASVTYVATRTAAGGYSDDINKQHDTTNAYGVDTGRQHSSGGYDGDTNKHHGTTGGYNDDTNRHHGTTGVYGIDTDRQQHGTTGGYAGDTGRQHGNIGGPYYGTNTADTGTGPRSGTTGGTYGGTGGTDYGTTGGTYGGSGTGYGVNTGGAHTEAGYRKEHRQHDQSHGDQNEKKGIMDKIKEKLPGGHSDK
Glycine max	Glyma.07G090400-Y2K	ND		243	MASYQKHYDDQGRKVDEYGNVERQTDEYGNPVHATSVTYVATKSVGGYNDDTNKQHDTTGVYPEKDTGRHHFGRGYDGDTNKQQHDAIGVYPGIDIGRDHETTGVYGLNTDRHHGTTGVNPGIDTHNQQHGTTGGYAGDTGRQHGNTGGLYYGTDTADTGTGPRSGNTGGTGVYGGTGGTDYGTAGGTGYGGSGTGYGINTGGAHTEAGYGKEHRQHEQSHGGQHEKKGILDKIKEKLPGGHSDK
Malus domestica	MdDHN5-YK4	ND		256	MAHYQNQHGATPQETDQFGNPIQHGTTGATGFGTATTGYGTTNDTYTGGQQQQKRGMMDKIKDKIPGTGGARRADDHYSSHAHTGIGTTGTGYGTTAAPSTGGEYERRGLMDKIKDKIPGTGGRTDHPTPYGGTTYTGEHQHEGIADKIKDKIPGTGHRDDAYSSHTTSTTAPYGGTTGQHHSTTGQHHSTTAPYGGTTGQHHSTTGQHHSTTAPYGGTTGQHHSTTTPYGGTTGQHHEKKGIMDKIKEKLPGGHH
Malus domestica	MdDHN6-YK3	4.70<br>7.78<br>0.84	11-55<br>98-133<br>156-171	190	MANYQSQYGTKPQDTDEYGNPIQQGTTGGFGSATTGTGYGTTETPYTGGQQQQERGTGMTGSGYGTTDAPYTGGQQQQERGMMDKIKDKIPGTGGGRRDDDPYSSNAQTGTGMTGTGYGTTDAPYTGGEYEERGMMDKVKDKIPGTGQRDETYSSKTASTTAPYGGTTGEHQEKKGAMDKIKEKLPGGRH
Medicago falcata	ABX80061.1	ND		307	MSQYQQGGDQTGRVDEYGNPLSQGQVDQYGNPISGGGMTGATGHGHHQQHHGVDTGYGAHTGSGGAPTGGGYGGNDIDGTGYGTGTGTGTTGYGATGGGTGHDNRGVVDKIKEKIPGTGTGTGTGRGTTGYGDTGVGQGTTGVGHQQHGENRGVVDKIKEKIPGTGTGTGTGHGTTGYGATRTGTGHGTTGYGATDVGHGTTGVGHQQHGDNRGAVDKIKEKIPGTEQNIYGTGTGTGTGTGHGTGTGTGHGHGTTGYGSTGQEYGKEGHHGHDEQHLGEKKGIMEKIKEKIPGTGSCTGHGQTKP
Medicago sativa	*AAL15651.1*	ND		330	MSQYQQGGDQTGRVDEYGNPLSQGQVDQYGNPISGGGMTGATGHGHHQQHHGVDTGYGAHTGSGGAHTGGVGGYGGTDIDGTGYGTGTGTGTTGYGATGGGTGHDNRGVVDKIKEKIPGTGTGTGTGHGTTGVGHQQHDGTYGTGTGTGYGTTGVGHQQHGDTYGTGTGTGNWNWNEVGHGTTGYGATDVGHGHQQHGENRGVVDKIKEKISGTGTGTGTGHGTTGYGAGGVGHGHQQHGEKGVVDKIKEKIPGTEQNTYGTGTGTGTGHGTTTGYGSTGQEYGREGHHGHDEQHLGEKKGIMEKIKGEALVLDHVLDMDKLNHNIYGCMHLI
Medicago truncatula	Medtr6g084640-Y2K3	ND		312	MSQYQQGYGDQTRRVDEYGNPLTSQGQVDQYGNPISGGGMTGATGHGHGHHQQHHGVGVDQTTGFGSNTGTGTGYGTHTGSGGTHTGGVGGYGTTTEYGSTNTGSGYGNTDIGGTGYGTGTGTGTTGYGATGGGTGVGYGGTGHDNRGVMDKIKEKIPGTDQNASTYGTGTGYGTTGIGHQQHGGDNRGVMDKIKEKIPGTDQNQYTHGTGTGTGTGYGTTGYGASGVGHQQHGEKGVMDKIKEKIPGTEQNTYGTGTGTGHGTTGYGSTGTGHGTTGYGDEQHHGEKKGIMEKIKEKLPGTGSCTGHGQGH
Phaseolus vulgaris	Phvul.004G158800-YK	ND		259	MASYQKQYEDEQGRKIDEYGNVVKTTDEFGNPVHSTSVTYIATTGTGRVTDDTNKQHDTSNTSNLYGVNTARQHGTTGGFTGDTGRQHGTTGGFTGDTGRQHGTTGGFTGDTGREHGTTGGFTGDTGRQHGTTGGFTGDTGRQHGTTGGFTGDTGRNHKTTGGFTGDTGRQHGTTGGLTGDTGRQHGTTGGFAGDTAVGGGPYVGDNTADTGTGPRSGTGGGYGSGTGAGYGREHGNEEKKGIMDKIKEKIPGVHSDNK
Physcomitrella patens	PpDHN Y11K	4.30	514-540	554	MNQYGREQQDTGLVGSGTGHRDEYGNPRQEGIMDKVKNAVGMGPSSGTGYNNQPGYDNYGNPRQEGLVDKAKDAVGMGPSLGTGYNNQPGYDSYGNREGIVDRAKDAVGMGPNSGTGYNNQPGYDNYGDRRHEGLADRAKDAVGMGPNSGYNHQPGYDNYGNREGIVDRAKDAVGMGPNSGTGYNNQPGYDSYGTSTRRQEGLVDRAKDAVGMGPNSGTGYNNQPGYDNYGNPRREGVVDRAKDAVGMGPNSGYNNQPGYDNYGNREGIVDKAKDAVGVGPHSGTGYHNQPSYDNYGNPRQEGIVDRAKDAVGMGPNSGTGYNNQSDYDSYGNPRHEGMLDKAKDDF[illegible]
Pisum avestivum	AAB51380.1	ND		225	MSQYQNQYGAQTRKTDEYGNPVNQVDQYGNPISGGGLTGEAGRQHYGTTGGATDHGHGHGQQHRGVDQTTGYGTHTGGVGGYGTKPEYGSTNTGSGYGTGTGYGGSGTTEYVREEHHGDKKGVMDKIKEKIPGTEQSRTNTDGTGYGSTGYGASGGGIGNTGQEYVREERHVHPGDKKHGSAGQEYVREEHHVHPGDKKHGSAGQEYVKEERRGIGNTGQEYVREERHVDHGEKKGIMDKIKEKLPGTGGCTGH
Pisum avestivum	*P28641.1*	1.6	86-138	232	MSQYQNQYGAQTGMTDEYGNPVNQVDQYGNPISGGGLTGEAGRQHYGTTGGATGQGHGHGQQHRGVDQTTGYGTHTGGVGGYGTKPEYGSTNTGSGYGTGTGYGGSGTNPDYGSTNTGSGYGTGTGYGGSGTTEYVREEHHGDKKGVMDKIKEKIPGTEQSRTNTDGTGYGSTGQGYVREQQHGTGYGSTGQGYVREQQDVHGDEQHGEKKGIMEKIKEKLPGTGACTGH
Prunus dulcis	AAY98416.1	ND		272	MAHFGSTPEPTKTDEYGNPVHHTTGTGRTDEFGNPVQHGVADTGYGTGAGYATHTKPGVVEHHGVPAVLHSKDDQYSRDTQTTTGGYGGDGYTGGEHQEKKGLLGQLQDKLPGGNQDGQYSHDTQTTTGAYGGAGYTGGEHPEKKGIIGQVKDKLPGGQKDDEYCRDTHPTTGAYGGTGYTGGEHHEKKGIIGQVKDKLPGGQKDDQYCRDTHPTTGAYGGAGYTGGEHQEKKGIIGQVKDKLPGGEKXDQYSHDXHKTTGAYGGAGYTEM
Prunus persica	*XP_007202596.2*	ND		457	MAHFGSTPEPTKTDEYGNPVHHTTGTGRTDEFGNPVQHGVADTGYGTGAGYGTHTKPGVVEHHGVPAVLHSKDDQYSRDTQTTTGGYGGDGYTGGEHQEKKGLLGQLQDKLPGGNKDGQYSHDTQTTTGAYGGAGYTGGEHHEKKGIIGQVKDKLPGGQKDDQYCRDTHPTTGAYGGTGYTGGEHHEKKGIIGQVKDKLPGGQKDDQYCRDTHPTTGAYGGAGYTGGEHQEKKGIIGQVKDKLPGGQKDDQYSHDTHKTTGAYGGAGYTGDDTREKKGGQKDDHYSHDTHPTTGAYGGAGYTGDDTREKKGIIDQVKDKLPGGQKDDHYSHETHPTTGAYGGAGYTGDDTREKKG[illegible]
Prunus persica	ppa009997m.g-YK2	0.69<br>7.88<br>11.97<br>1.36	1-51<br>208-249<br>176-208<br>126-145	268	MANYQNQYGATAQSTNDYGNPLTNYTGSEPQSTNEYGATDEYGNPVTTYTRSEPHYTDEYGNPVRERGMMDKIKEKLPGTHDPYSSHSDTTYPTAPYGGTYGTGLEQHHGMMNKMPGTDPYSSHSHTTGTTAPYDGTYGTGTEYRQHKGTMDRIKDKLTGTGGHRVDDPYSSHSHKTATTTPYGGTTTYAYGDSTTTPSPYGGTTTHSPYGGTTTTAYGDTTTTPSAYGGTTTTPSAYGGTATTTYPREKKGIMDKIKEKLPGGHHY
Pyrus pyrifolia	ACK37880.1	7.78<br>9.82	93-128<br>148-165	184	MANYQTKYGTTPQDIDEYGNPIQQGTTGGLGGATTGTAYGATGGQQQQERGTGMTGSGYGTTDAPYTGGQQQQERGMMDKIKEKIPGTGGGRRDDDPYSSNAQTGTGMTGTGYGTTDAPYTGGEYEERGMMDKVKDKMPGTGQRDETHSSQTTTTTPYGGTTGEHQEKKGAMDKIKEKLPGYRH
Sarracenia purpurea	SapurV1A.2659s0010-<br>YK10	ND		556	MAGIMQKIEETFGGKKEEQRKGEPQPGLGQQGHNTQDEYGQQGYNAQGEHKEGFAGNIKDKIPGGGGVGSGGMHKGETQGGYVQQGYNTQGGYGQQKYNVEGEHKEGIIDKIKDKISGGGGGMHKGETQGGYGGQEHNAQGEHKEVFVVNVKDKIPGGGSVGGGGIHKGETQGGYGGQRHNTQGGYGQQGYNAQGEHKEGIVDNIKDKIPGGGSHDGVGGLGDGDGGGGGMHTGETQGGYMQQGQNTQGGYGQQGYNTQDECKEGFVDNIKDKIPGGVGGGVGGGGMHKGETQGRYGQQGHNTQGGYGQQRYNAPGERKEGFVDNIKDKIPGSGGGNVGGGGMHK[illegible]
Sarracenia purpurea	SapurV1A.5492s0010-<br>YK3	10.21<br>0.92	90-113<br>6-19	175	MASTIRDEQGNPIQLTDEHGSPVHLTDEHGDSVQIIGIATTKQPPTSFRYGSSDQVTGTGLWSSTAKSEDAMKGTDIHETGQRFRGGHKKEEQTEISSTSILGSPEDDGQGGRKGLKQKIKEKLTGGKHGEEHEHTVEVDTTTTARPAGEHYQEHQKKGVVEKIKEKLPGHHGHH
Vicia monantha	BAH70483.1	ND		195	MSQYQNQYGAAQTGMTDEYGNPVNQLDQYGNPVSGGGFTGEAGRQHFGTTGAAGHGHGQQHRGDDQTTGYGTNTGGVGGYGTKPEYGSTNTGSGYGTGTGYGGTGTTEYVREEHHGDKKGVMDKIKEKIPGTEQSRTNTEGAGYGTTGYGASGGGMGNTGQEYVREERHVDHGEKKGIMDKIKEKLPGTGGCTGH

KS-Type

Specie	Access number/Name	PEST score	Position	Protein size	Sequence
Aquilegia coerulea	Aquca_004_00603-KS	ND	93		MAGIMNKIGDALHIGGNKEEQKQGEAHKTEIKHGETAHKPEQKEGFVDKIKDKITGQGPHKTEGGGEKKKKKEKKKKEGDHGHDGSSSSSDSD
Arabidopsis lyrata	AL1G64750	ND	98		MAGLINKIGDALHIGGGNKEGEHKKEEEHKKHVDEHKSGEHKEGIVDKLKDKIHGGEGKSHDGEGKSHDGEKKKKKDKKEKKHHDDGHHSSSSDSDSD
Arabidopsis thaliana	AT1G54410-KS	ND	98		MAGLINKIGDALHIGGGNKEGEHKKEEEHKKHVDEHKSGEHKEGIVDKIKDKIHGGEGKSHDGEGKSHDGEKKKKKDKKEKKHHDDGHHSSSSDSDSD
Boechera stricta	Bostr.13404s0009	ND	99		MAGLINKIGDALHIGGGNKEGEHKKHEEEHKKHVDEHKSGEHKEGIVDKIKDQIHGGEGKSHDKEGKSHDGEKKKKDKKEKKHHDDGHHSSSSDSDSD
Brachypodium distachyon	Bradi1g13330-KS	ND	100		MAGIIHKIEEKLHMGGGSDHKDEHKKEEEHKKKDGEHKKDGEHKEGMMEKIKDKITGEHGDGDKSSGDHKEKKDKKKKEKKHGEGHKKEDGHSSSSDSD
Brassica oleracea var. capitata	Bol042507	ND	95		MAGIINKIGDALHIGGGNKEDEHKKEEHKKHADEHKSGEHKEGIVDKIKDKIQGGEGHSSGDHKHDGEKKKKDKEKKHHHDGHHSSSSDSDSD
Brassica rapa	Brara.C04012	ND	95		MAGIINKIGDALHIGGGHKEDEHKKEEHKKHADEHKSGEHKEGIVDKIKDKIHGGEGHSSGDHKHDGEKKKKDKEKKHHDDGHHSSSSDSDSD
Capsella grandiflora	Cagra.27207s0001	ND	100		MAGLINKIGDALHIGGGNKEGEHKKHEEEHKKHVDEHKSGEHKEGIVDKIKDKIHGGEGKSHDGHEGKSHDGEKKKKKDKKEKKHHGDGHHSSSSDSDSD
Capsella rubella	Carubv10021201m.g	ND	100		MAGLINKIGDALHIGGGNKEGEHKKHEEEHKKHVDEHKSGEHKEGIVDKIKDKIHGGEGKSHDGHEGKSHDGEKKKKKDKKEKKHHGDGHHSSSSDSDSD
Carica papaya	evm.model.supercontig_<br>161.14-KS	ND	93		MSGIMHKIEDALHIGGRKKEEEHKGEHHGEHKGEHKGEHHGEHKGEHKEGIVDKIKDKIHGEGHEEKKKKKEKKHGEHGHDGHSSSSDSDSD
Daucus carota	DCAR_013481	ND	90		MSNIIHKVEEKLGLGGDHKDGKHHDDHKKSEHKEGIVDKVKDKVHGDKSGDHHGHGHGHGHGDGKKKDKKKKKHEDGHKHGDSSSGSDSD
Glycine max	Glyma.16G037900-KS	ND	112		MSGIIHKIEETLHVGGHKKEEEHKGEQHHGGEYKGEHHGEHSTEYKGEHHVGEHKPEHHGGEEHKEGFLDKIKDKIHGEEGGATAEGEKKKKKEKKKHEHGHDSSSSSDSD
Kalanchoe fedtschenkoi	Kaladp0098s0091	ND	87		MSGIINKIGDALHIGGDKKAEEHHKPEDHHKADGHKEGVVEKIKDKIHGEKSGEHHEGEKKKKEKKKKKEGEKKHHGGDSSSDSDSD
Kalanchoe fedtschenkoi	Kaladp0911s0009	ND	107		MSGIMNKIGDALHIGGDKKAEEHHKAEEHHKADEHHKADEHHKAEEGHKSGEHKEGLLDKIKDKIHGDGEKSGEHHEGEKKKKEKKKKKEGEKKHDGGDS
Linum usitatissimum	Lus10020271-KS	ND	93		MSGIIHKIEETLHIGGGDDHKDKDKDKHHGHGGDHKHSSGDHKEGIVDKMKDKIHGEDHHDHHGHDGEKKKKKKEKKKHEDGHESSSGSDSD
Manihot esculenta	cassava4.1_019934m.g-<br>K2S	ND	102		MSGIIHKIEETLHIGGNKKEEHKGESHGHGDHKDHKDHKDHKGDHHKGEGEHKEGIVDKIKDKIHGDHEHGDGDKKKKKKEKKKHEHGHDHDGHSSSSSDSD
Medicago truncatula	Medtr7g086340-K2S	ND	121		MAGIMNKIGDALHIGGDKKEGEHKGEQHGHVGGEHHGEYKGEQHGFVGGHGGEHKGEQHGFGHGDHKEGHHGEEHKEGFVDKIKDKIHGEGADGEKKKKKEKKKHGEGHEHGHDSSSSDSD
Medicago truncatula	Medtr6g027810-K2S	ND	100		MAGIINKIGEALHVGGGHKKEDEHKGEKSHDDKHKGEKSHDDKHKGEHKEGIVDKIKDKIHGGDGEGHEHKGEKKDKKKKDKKKKEHGHDHDSSSSSDSD
Mimulus guttatus	Migut.J00768-K2S	ND	119		MAGIIHKIEEKLGLGGGHKEEEKKAEHSYAEQIKPDHHGEKKAEHGYGGGEKKPEEHKEGLIEKIKDKIHGGDEGESGECKGEKKDKKKKDKKEKKKHCDGDGEDKHGGSSSDSGSDSD
Oryza sativa	OsLIP5-KS	ND	92		MAGIIHKIEEKLHMGGGEHKKEDEHKKEGEHHKKDGEHKEGVVEKIKDKITGDHGDGGEHKEKKDKKKKEKKHGEEGYHHDGHSSSSSDSD
Oryza sativa	OsWS1724-KS	ND	92		MAGIIHKIEEKLHMGGGEHKKEDEHKKEGEHHKKDGEHKEGVVEKIKDKITGDHGDGGEHKEKKDKKKKEKKHGEEGHHHDGHSSSSSDSD
Panicum virgatum	Pavir.Ia01114-KS	ND	101		MSGIIHKIEETLHMGGDHKKEEEHKKAEAHKKAEEHHKKDGEHKEGIVEKIKDKITGEHGDKSGEHKDDKDHKEKKDKKKKEKKHGEGHHDGHSSSSDSD
Populus trichocarpa	PtrDHN-6-KS	ND	189		MAGIMHKIEETLNIGGKKDERKGETQGGYNQQDHRDGAQGQRKEGFVDQMKDKIPGGGGGVHKGETQGGYNHQEQGGYNQQQHRGGAQGERKEGFVAQMKDKLPGGGGGMTQGGYNQQEHRGGAQGGYNQQEHRGDAQGERKEGFVDKIKGKIPGGGGGSGVRGEGGEKKKKKDKKKNEDGHSSSSDSD
Populus trichocarpa	PtrDHN-10-KS	ND	108		MASCQCSKPVEHPCNQDQKSHSSGQKVEKQAEGGVVKTGTRSSSQSHSPGSTNGMTPAPACNANKRGERKKGLFQRIKDGISGHSDGGGSSSSSESESDDEKCGKRKN
Populus trichocarpa	Potri.013G062400-KS3	ND	239		MNEDLRDLFLILNVAARGSFHGAYKCGTRFIHHFISKAISKREKRRKKYTMAGIMHKIEETLNIGGKKDERKGETQGGYNQQDHRDGAQGQRKEGFVDQMKDKIPGGGGGVHKGETQGGYNHQEQGGYNQQQHRGGAQGERKEGFVDQMKDKLPGGGGGMTQGGYNQQEHRGGAQGGYNQQEHRGDAQGERKEGFVDKIKGKIPGGGGGSGVRGEGGEKKKKKDKKKNEDGHSSSSDSD
Ricinus communis	30072.t000037-KS	ND	96		MAGIMHKIEETLHIGGHKKEEHKGESHDQHKHDQHAHGDHPKPEGEHKEGFMDKIKDKIHGDEKGHEHGHGESGEKKKKKEKKKHEHGHSSSSDSD
Sorghum bicolor	Sobic.001G149500	ND	101		MSGIIHKIEETLHMGGDHKKDDEHKKEEHKKAEEHHKKDGEHKEGIVEKIKDKITGEHGEKSGEHKDKDHKEKKDKKKKEKKHGEGHDHDGHSSSSSDSD
Trifolium pratense	Tp57577_TGAC_v2_ge<br>ne29874	ND	100		MSGIINKIGETLHIGGGHKKEDEHKGEKSHDDKHKGEKSHDDKNKGEHKEGIVEKIKDKIHGGEDHESKGKGEKKDKKKKDKKKKEHGHDHDSSSSSDSD
Zea mays	Zm00008a004788	ND	103		MSGIIHKIEEKLHMGGDHKNKEEEHHKKAEEHHKKEEGEHHKKDGGEHKEGIVEKIKDKITGEHGDKSGDHKEKKDKKKKDKKKHGEGHDHDGGHSSSSSDSD
Zea mays	GRMZM2G169372-KS	ND	209		MPLTMIPCRRLLETDAMLRRAGRKKRKVGCFACTSSWHAEQLVHYLLFSAITSFLSDWPSIPSINTGRRRRANQDHRRVAQSIIAVSLLREQRKRARRAVAMSGIIHKIEEKLHMGGDHKNKEEEHHKKAEEHHKKEEGEHHKKDGGEHKEGIVEKIKDKITGEHGDKSGDHKEKKDKKKKKHGEGHDHDGGHSSSSSDSD
Zea mays	GRMZM2G448511-KS	ND	108		MSGIIDKIQETLHIGGDHKEEHEHKKGEEHHKKDDGEHHKKDDGEHKEGIVEKIKDKITGEHGDKSGDHKDKDHKEKKDKKKKEKKHGEGHDHGDGDGHSSSSSDSD
